# Research advances in connective tissue disease-associated pulmonary arterial hypertension with an emphasis on the Chinese population

**DOI:** 10.3389/fmed.2026.1811378

**Published:** 2026-05-04

**Authors:** Baocheng Liu, Zihao Wang, Xin Li, Qiyao Liu, Jingyu Zhu

**Affiliations:** 1 Heze Traditional Chinese Medicine Hospital Affiliated with Shandong University of Traditional Chinese Medicine, Heze, Shandong, China; 2 Department of Gastroenterology, Jinan Central Hospital Affiliated to Shandong First Medical University, Jinan, Shandong, China

**Keywords:** clinical features, connective tissue disease, Han Chinese populations, prognosis and treatment, pulmonary arterial hypertension

## Abstract

Connective tissue disease-associated pulmonary arterial hypertension (CTD-PAH) is a severe pulmonary complication of connective tissue diseases (CTDs) that occurs in approximately 30% of adult patients with pulmonary arterial hypertension (PAH) and is associated with a substantial increase in mortality risk. Its incidence varies widely across regions: in Europe and North America, systemic sclerosis-associated PAH (SSc-PAH) predominates, whereas in China, systemic lupus erythematosus-associated PAH (SLE-PAH) and Sjögren’s syndrome-associated PAH (SS-PAH) are more common. However, contemporary data on clinical characteristics and prognosis in Han Chinese populations are limited, indicating a need for targeted studies to optimize diagnostic and therapeutic strategies and improve patient outcomes. This review summarizes the epidemiology, pathogenesis, clinical features, and prognostic factors of CTD-PAH and discusses potential implications for clinical management.

## 1 Introduction

Connective tissue diseases (CTDs) are chronic inflammatory disorders characterized by immune-mediated, multisystem and multiorgan autoimmune damage (1). The lungs are among the organs most frequently affected by CTDs because of their abundant connective tissue and dense vascular network. Pulmonary involvement spans multiple structures and pathological processes and mainly presents as interstitial lung disease, pleuritis, bronchiolar disease, pulmonary capillary lesions, and connective tissue disease-associated pulmonary arterial hypertension (CTD-PAH).

Pulmonary hypertension (PH) is defined as elevated pulmonary arterial pressure resulting from any cause and commonly occurs in patients with pulmonary arterial hypertension (PAH), left heart disease, chronic hypoxia, hematological disorders, metabolic conditions and immune–inflammatory diseases. On the basis of pathophysiology and clinical presentation, the World Health Organization (WHO) classifies PH into five groups: (1) arterial PH; (2) PH due to left heart disease; (3) PH due to lung disease and/or hypoxia; (4) chronic thromboembolic PH; and (5) PH with multifactorial and/or unknown etiology (2). Currently, echocardiography is an important noninvasive screening tool for PH. According to the latest ESC/ERS guidelines, echocardiography can be used as a method for assessing the probability of PH (3): a peak tricuspid regurgitation (TRV) velocity greater than 3.4 m/s indicates a high risk of PH regardless of whether other ultrasonic abnormalities are present. When the TRV is within the critical range of 2.9 to 3.4 m/s, other PH-related indicators, such as right ventricular dilation, abnormal morphology of the interventricular septum, and elevated estimated pulmonary artery systolic pressure, must be considered to increase screening accuracy. When the TRV is less than or equal to 2.8 m/s and no accompanying signs are observed, the probability of PH occurrence is relatively low, and further invasive examinations are not recommended (Tables 1, 2). Currently, right heart catheterization (RHC) is the gold standard for confirmatory diagnosis of PAH. On the basis of hemodynamics, precapillary PH, also known as PAH, is defined by a mPAP >20 mmHg, a pulmonary vascular resistance (PVR) value >2 Wood units, and a pulmonary arterial wedge pressure (PAWP) ≤ 15 mmHg (4). This change in diagnostic criteria may result in earlier detection of PH. Certain populations with PH may benefit from earlier initiation of therapy (4).

**Table 1:** Possibility of diagnosing PH by echocardiography in patients with suspected PH.

Peak velocity of tricuspid regurgitation	Other echocardiographic signs supporting PH are present	Probability of PH
≤ 2.8 m/s or not detectable	No	Low
≤ 2.8 m/s or not detectable	Yes	Moderate
2.9 ~ 3.4 m/s	No	Moderate
2.9 ~ 3.4 m/s	Yes	High
>3.4 m/s	Yes/no	High

Reproduced from Shah R. K. Echocardiographic evaluation of pulmonary hypertension. Journal of the Indian Academy of Echocardiography and Cardiovascular Imaging, 2018, 2 (2): 95–105. doi: 10.4103/jiae.jiae_6_18. This article is licensed under a Creative Commons Attribution License, which permits unrestricted use, distribution, and reproduction, provided that the original work is properly cited (152).

**Table 2:** Echocardiographic signs supporting the diagnosis of PH.

Part	Condition
A: Ventricle^a^	Right ventricular internal diameter / Left ventricular internal diameter > 1.0; Interventricular septum flattening (systolic and/or diastolic left ventricular eccentricity index > 1.1)
B: pulmonary artery^a^	Doppler right ventricular outflow tract acceleration time <105 ms, and/or mid-systolic notch; Early diastolic pulmonary regurgitation velocity > 2.2 m/s; Main pulmonary artery diameter > 25 mm
C: Inferior vena cava and right atrium^a^	Inferior vena cava diameter >21 mm with collapse of the inferior vena cava during inspiration (collapse rate of the inferior vena cava <50% during deep inspiration or <20% during quiet breathing); Right atrial area at end-systole >18 cm^2^

^a^At least two of A, B, and C are met.

Reproduced from Zhang X., Zhao J. L., Ding F., Yang J., Wang J., Zeng X. F., et al. Recommendations for the diagnosis and treatment of connective tissue disease -associated pulmonary arterial hypertension in China. Chin J Intern Med. (2022) 61: 1206–1,216. doi: 10.3760/cma.j.cn112138-20220309-00164. This article is licensed under a Creative Commons Attribution License, which permits unrestricted use, distribution, and reproduction, provided that the original work is properly cited (118).

PAH is a group of diseases characterized by progressive increases in pulmonary vascular resistance and gradual occlusion of the pulmonary vascular bed, frequently leading to right heart failure and death (5). CTD is closely associated with PAH, and CTD-PAH occurs in patients with various CTDs, including systemic sclerosis (SSc), rheumatoid arthritis (RA), systemic lupus erythematosus (SLE), polymyositis/dermatomyositis (PM/DM) and mixed connective tissue disease (MCTD) (6–8). Previous studies have shown that CTD-PAH is the main cause of low quality of life in CTD patients and is closely related to increased mortality (9, 10). This review provides a systematic examination of research on CTD-PAH because of its clinical implications in the Han Chinese population. To strengthen the research foundation and ensure the scientific rigor and comprehensiveness of the review, literature from multiple databases was retrieved.

A multi-database search strategy was adopted for literature retrieval, covering four major English databases: MEDLINE, EMBASE, Cochrane, and Google Scholar. The search was conducted from January 2013 to December 2025. Moreover, the reference lists of the retrieved articles were manually reviewed to uncover potentially relevant studies for supplemental evidence and to avoid omissions. The titles, abstracts, and medical subject headings (MeSHs) of the retrieved articles were searched for terms and abbreviations including pulmonary hypertension (PH), pulmonary arterial hypertension (PAH), systemic lupus erythematosus (SLE), connective tissue disease (CTD), systemic sclerosis, primary Sjögren’s syndrome (pSS), dermatomyositis (DM), polymyositis (PM), mixed connective tissue disease (MCTD), epidemiology, pathophysiology, biomarkers, management, treatment, and prognosis. This strategy ensured comprehensive coverage of the core research content for this review while also considering studies involving both foreign populations and Chinese Han populations with CTD-PAH, thereby providing sufficient literature support for the comparative analysis of the characteristics of the two groups. Two researchers (BL and ZW) independently retrieved the literature to minimize human bias. The inclusion criterion was clinical studies (including clinical investigations and clinical meta-analyses); experimental studies were used only to supplement the explanation of pathophysiological mechanisms. The exclusion criteria were the inability to obtain the full text, non-English publications, and conference abstracts. Literature was screened in three steps. First, an initial search based on the search terms yielded 1,429 articles. Second, on the basis of the titles and abstracts of the articles, 56 duplicate articles and 1,071 articles irrelevant to CTD-PAH were excluded, resulting in the inclusion of 302 articles in the full-text academic review. Finally, in accordance with the inclusion criteria (clinical studies, full text available, and related to CTD-PAH) and exclusion criteria (basic experimental research, conference abstracts, and non-English articles), 213 articles that did not meet the requirements were further excluded. Ultimately, 89 articles were included, including 12 systematic reviews and meta-analyses of clinical data, 47 clinical studies on foreign populations, and 30 clinical studies on Chinese Han populations. These articles fully met the needs of this review for the analysis of the characteristics of CTD-PAH and the exploration of treatment mechanisms across different populations.

Using the results of the comprehensive literature search mentioned above, this review aims to summarize the pathophysiological mechanisms of CTD-PAH, highlighting the comprehensive diagnostic strategies used on the basis of multiple biomarkers, and providing a detailed overview of targeted treatment and management methods related to improving long-term patient prognosis to provide a reliable reference for clinical practice and subsequent related research.

## 2 Epidemiology of CTD-PAH

**Figure 1:**
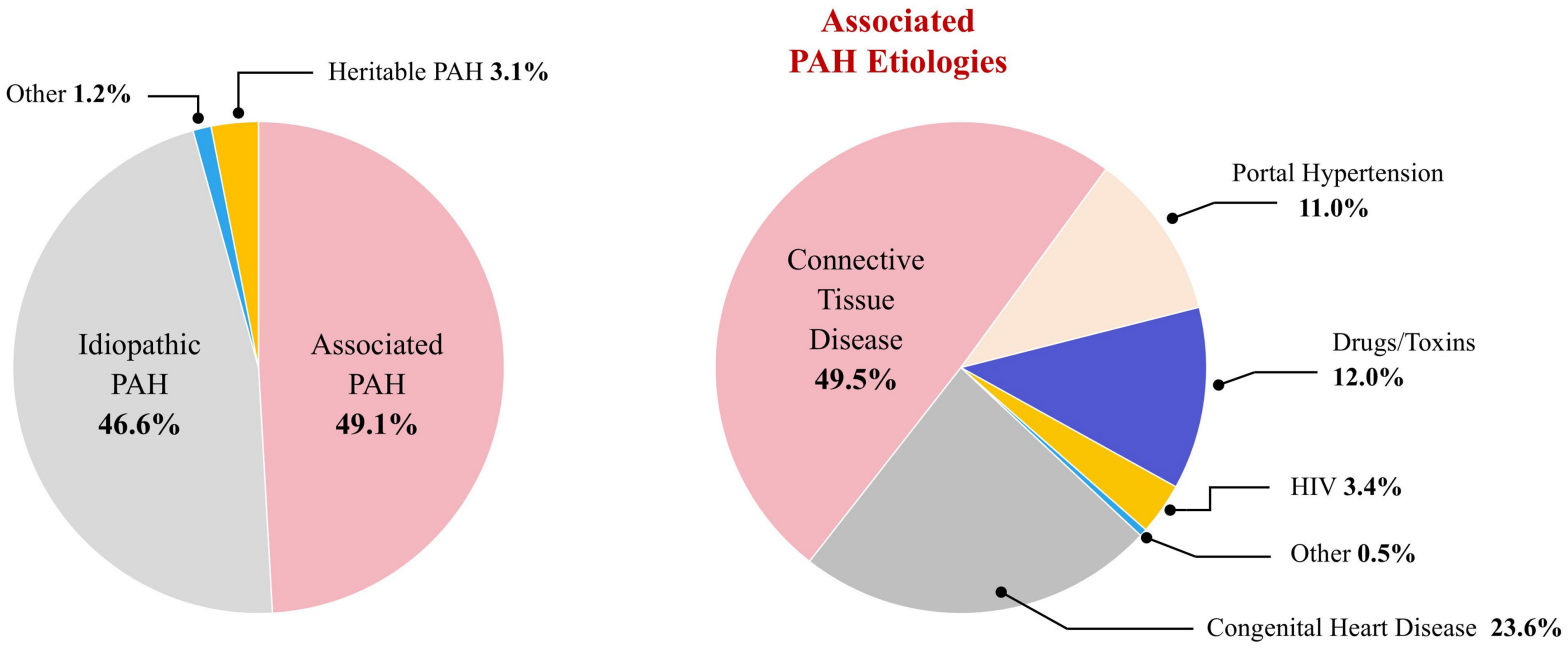
Proportion of patients with CTD-PAH in the overall PAH. This figure is the original work of the authors and has not been published previously or submitted elsewhere.

Epidemiology and risk factors of connective tissue disease-associated pulmonary hypertension vary by disease subtype (6, 89, 148, 153, 154, 155, 156). The incidence of CTD-PAH varies slightly across regions. Epidemiological studies from Europe and the United States have reported that approximately 8 to 14% of patients with SSc develop SSc-PAH (11) (Table 3). Among all PAH cases, CTD-PAH represents the second most common subtype following idiopathic pulmonary arterial hypertension (IPAH), comprising approximately 30% of all adult PAH cases (Figure 1).

**Table 3:** Summary of the prevalence, risk factors, and prognostic markers of CTD-PAH.

CTD	Prevalence	WHO PH groups	Factors associated with PAH	Factors associated with shorter survival	Good prognostic markers
SSc (6, 148, 153)	8–14%	1, 2, 3, 4, or 5	Limited SSc + Anticentromere Ab + Anti-U3 RNP ↑Telangiectasias	ge > 50 years Male sex Lower GFR HLA DRw6 and HLA DRw52 Lung disease	+ Anti-U1 RNP
SLE (89, 148)	0.1–2%	1, 2, 3, 4, or 5	Raynaud’s phenomenon Digital vasculitis High disease activity + Anti-U1 RNP Anticardiolipin IgG Ab ↑Uric acid	Thrombocytopenia Thrombosis Pulmonary vasculitis Raynaud’s phenomenon Anticardiolipin Ab	Younger age ↑DLCO
MCTD (154)	1–3%	1	ILD Pericarditis Thrombocytopenia + Anti-Sm antibodies		Younger age ↑DLCO
Sjogren’s disease (155)	0.2%	1	Young age ↓Sicca manifestations + Anti-U1 RNP + Anti-SSB Ab	↑Damage index	
IIMs (156)	0.3%	1 or 3	DM Skin involvement Peripheral microangiopathy + Anti-SSA Ab		

Ab, antibody; anticentromere Ab, anticentromere antibody; anti-Sm antibodies, anti-Smith antibodies; anti-SSA Ab, anti-Sjögren’s syndrome-related antigen A antibody; anti-SSB Ab, anti-Sjögren’s syndrome-related antigen B antibody; anti-U1 RNP, anti-U1 ribonucleoprotein antibody; anti-U3 RNP, anti-U3 ribonucleoprotein antibody; anticardiolipin IgG Ab, anticardiolipin immunoglobulin G antibody; DLCO, diffusing capacity of the lungs for carbon monoxide; GFR, glomerular filtration rate; HLA, human leukocyte antigen; IIMs, idiopathic inflammatory myopathies.

WHO PH groups, World Health Organization pulmonary hypertension groups.

Reprinted from Alturaif N., Alduraibi F. K. CTD-PAH: an updated practical approach to screening, diagnosis, and management. Therapeutic Advances in Respiratory Disease, 2025, 19: 1–12. doi: 10.1177/17534666251385674. This article is licensed under a Creative Commons Attribution License, which permits unrestricted use, distribution, and reproduction, provided that the original work is properly cited (11).

Data from the Chinese Systemic Lupus Erythematosus Assessment Registry (CSTAR) demonstrate that the prevalence of PAH among patients with SLE is approximately 3.89%. Given the large absolute number of SLE cases in China, SLE-PAH accounts for nearly 50% of all CTD-PAH cases, making it the most prevalent subtype of CTD-PAH in the Chinese population (12). In addition, pSS-PAH is the second most common CTD-PAH subtype in Asian and Chinese cohorts, representing approximately 19% of CTD-PAH cases. The prevalence of PAH in patients with pSS is approximately 0.22%. Nevertheless, given the high prevalence of pSS itself, PAH in this patient population remains clinically relevant despite its low disease-specific prevalence (13). Although patients with SSc have the highest prevalence of PAH among all individuals with CTDs, with reported rates ranging from 7.7 to 18.8% (14), the overall incidence of SSc is considerably lower than that of other common connective tissue diseases. Consequently, the absolute number of patients with SSc-PAH is smaller than that of patients with SLE-PAH or pSS-PAH (15).

PAH represents a severe pulmonary complication in patients with CTD. It not only significantly reduces survival and quality of life but also complicates clinical management, disease evaluation, and the implementation of therapeutic strategies (16). Compared with IPAH patients, those with CTD-PAH have a one-year survival rate of only 86% and substantially higher hospitalization rates (17).

Recent evidence indicates that compared with other CTD-PAH subtypes, SSc-PAH has a more aggressive clinical course, with a 3-year survival rate of less than 60% and a 5-year survival rate of approximately 50% (11). Within a UK cohort, the 3-year survival rate for SLE-PAH was 74%, whereas in a Chinese cohort, it was 88% (18). Furthermore, studies have indicated that the mortality rate of SSc-PAH patients is nearly fourfold greater than that of IPAH patients, with 1-year and 3-year survival rates of 87.8 and 48.9%, respectively, for SSc-PAH patients versus 95.1 and 83.6% for IPAH patients (19). Another study revealed that patients with concurrent interstitial lung disease (ILD) and PAH had poor prognoses, with 1-, 2-, and 3-year survival rates of 77, 48, and 35%, respectively (10).

However, not all cases of PAH in patients with CTD are pre-capillary PAH. Studies have shown that 17% of SSc-PAH patients have secondary left atrial disease, and all of these patients were confirmed to have postcapillary PAH by RHC (PAWP > 15 mmHg), possibly because of left ventricular dysfunction caused by myocardial fibrosis (3). A small number of CTD patients may develop PAH because of valvular lesions, such as endocarditis and significant mitral or aortic regurgitation, which can often be clearly observed on echocardiography or transesophageal echocardiography (20). ILD is a common complication of CTD, especially in patients with SSc or inflammatory diseases. Lesions of the pulmonary interstitium can damage the pulmonary capillary bed, and hypoxia induces capillary remodeling, thereby causing ILD-PAH (21). CTD patients are at high risk of venous thromboembolic events, especially those with positive antiphospholipid antibodies. Positive antiphospholipid antibodies are also important risk factors for the transformation of acute pulmonary embolism into chronic thromboembolic pulmonary hypertension (CTEPH) (1). CTD can also co-occur with conditions such as pulmonary artery stenosis and pulmonary small vein occlusion; however, these conditions are very rare in clinical practice (22).

## 3 Pathogenesis of CTD-PAH

**Figure 2:**
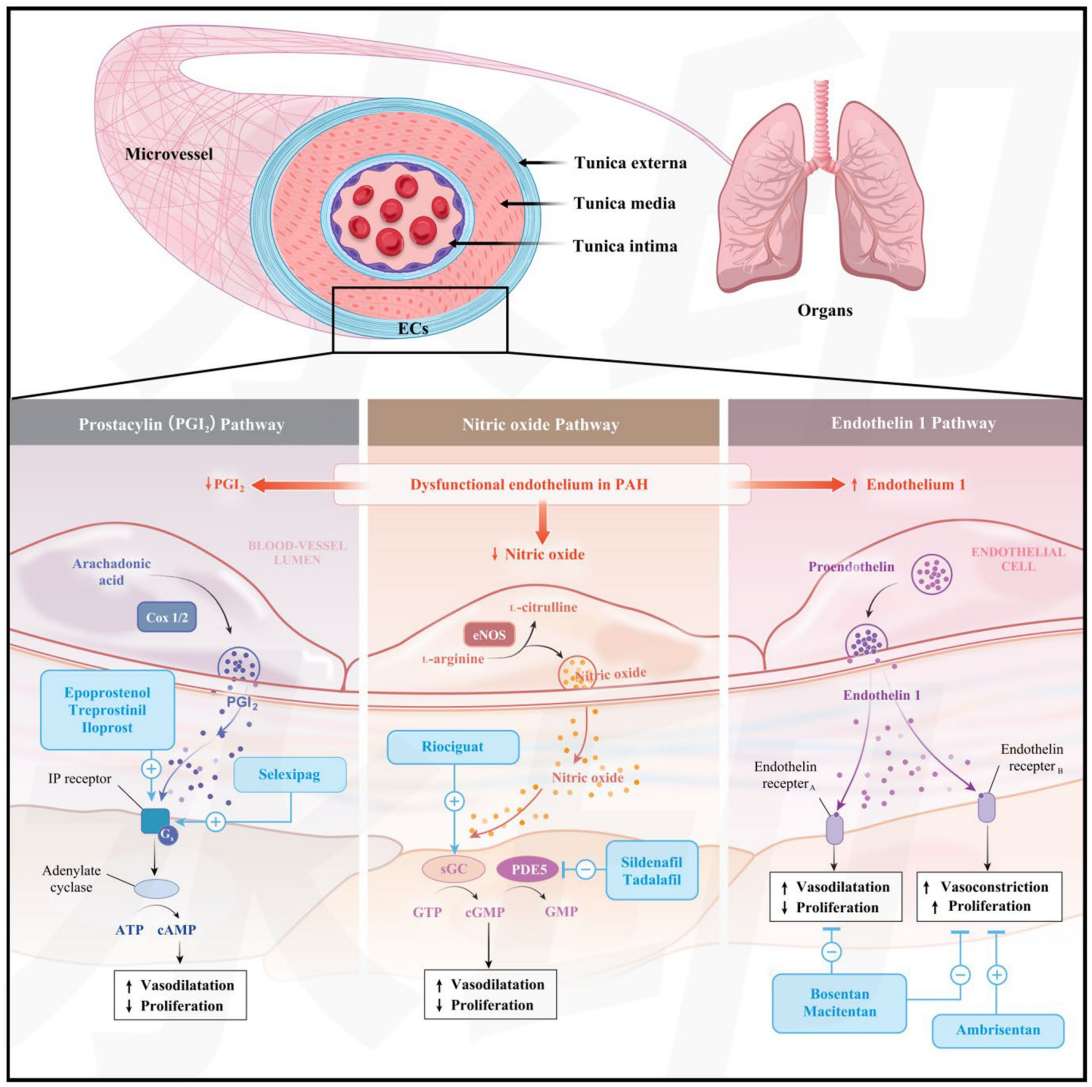
Three classic pathways of targeted therapy for PAH. This figure illustrates the core functional dysregulation of vascular endothelial cells in patients with PAH, characterized by reduced synthesis of PGI₂ and NO and increased secretion of ET-1. Additionally, the regulatory mechanisms and therapeutic targets of the three key pathways are presented. In the prostacyclin pathway, signaling deficiency is corrected by supplementation with PGI₂ analogs or activation of IP receptors; in the nitric oxide pathway, vasodilation is improved by enhancing the NO-sGC-cGMP signaling cascade or inhibiting cGMP degradation; and in the endothelin pathway, vasoconstriction and abnormal proliferation are suppressed through the blockade of ET receptors. AA, arachidonic acid; AC, adenylate cyclase; Cox 1/2, cyclooxygenase 1/2; eNOS, endothelial isoform of nitric oxide synthase; GS, G-protein-coupled receptor; GTP: guanosine triphosphate; NO, nitric oxide; ET-1, endothelin-1; PGI₂, prostacyclin. Reproduced from Hassoun P. M. Pulmonary Arterial Hypertension. N Engl J Med, 2021; 385 (148): 2361–2376. doi: 10.1056/NEJMra2000348. Used with permission from the Massachusetts Medical Society (149) and reproduced from Khakpour S, Wilhelmsen K, Hellman J. Vascular endothelial cell Toll-like receptor pathways in sepsis. *Innate Immun*. (2015) 21:827–46. doi: 10.1155/2012/854941. This article is licensed under a Creative Commons Attribution License, which permits unrestricted use, distribution, and reproduction, provided that the original work is properly cited (150).

Recent studies have shown that PAH develops because of progressive remodeling of small- and medium-sized pulmonary vessels. Proliferative lesions, including centripetal intimal thickening, pulmonary arteriole occlusion, small artery muscularization, and *in situ* thrombosis, constitute key pathological substrates underlying the progression of PAH and associated hemodynamic abnormalities (23). Proliferative lesions in pulmonary vessels predominantly localize to the bifurcations of pulmonary arterioles and are composed of multiple cell types, including mesenchymal cells, smooth muscle cells, and myofibroblasts, leading to the formation of a glomerulus-like structure. This distinctive pathological feature is typically seen in patients with severe PAH (24). Inflammatory and autoimmune processes play pivotal roles in the pathogenesis of CTD-PAH. Accumulating evidence suggests that both pulmonary vascular cells and infiltrating inflammatory cells may serve as major local sources of chemokines and cytokines, such as interleukin-6 (IL-6), IL-8, and tumor necrosis factor-*α* (TNF-α) (25). These mediators may contribute to excessive vascular cell vasoconstriction and abnormal fibroblast proliferation, thereby promoting pulmonary arterial remodeling (1, 26). These chemokines and cytokines may also act as potential biomarkers for disease progression and are correlated with unfavorable clinical outcomes (27). Autoimmune T-cell-mediated immune reactions and secondary immune dysregulation may promote the activation of pathogenic autoreactive B and T lymphocytes, which could further promote the initiation and progression of CTD-PAH (23, 28). Furthermore, endothelin-1 (ET-1) has been implicated in pulmonary vascular remodeling by stimulating mitosis in pulmonary vascular smooth muscle cells and fibroblasts, which may restrict dilatation of precapillary pulmonary vessels and contribute to elevated pulmonary capillary resistance (29, 30). Inflammatory and immune reactions may also induce focal vascular endothelial damage, downregulate the eNOS-NO-cGMP pathway, and reduce NO bioavailability, which in turn can promote pulmonary vasoconstriction and increase pulmonary vascular resistance (31, 32) (Figure 2).

**Figure 3:**
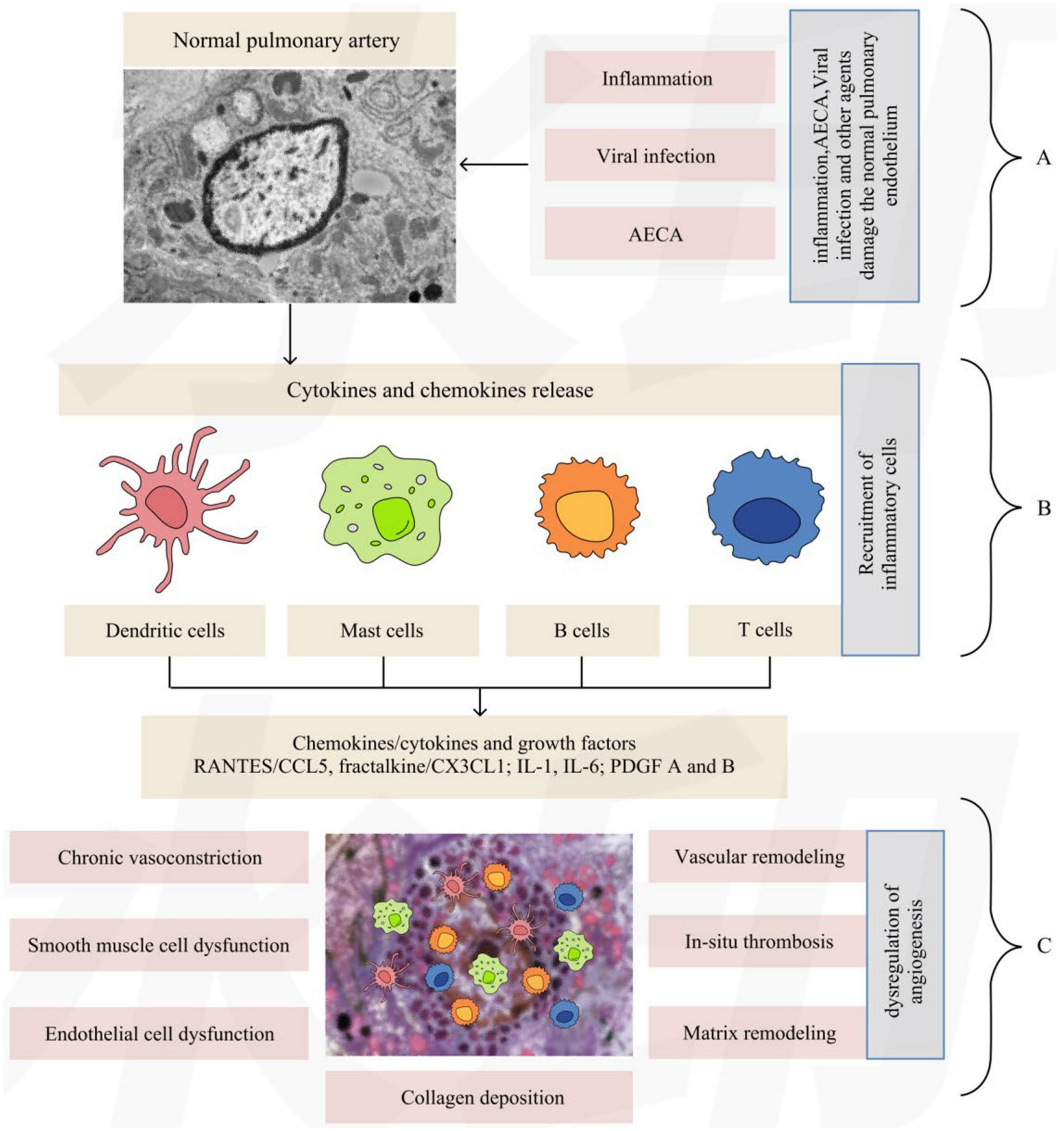
Role of inflammation and a dysregulated immune response in the development of PAH in patients with SLE. **(A)** Inflammation, AECA, viral infection, and other factors damage the normal pulmonary endothelium. **(B)** Endothelial cell injury leads to elevated concentrations of chemokines and cytokines, which in turn drive the recruitment of dendritic cells, mast cells, B cells, and T cells. **(C)** Dendritic cells, mast cells, B cells, and T cells infiltrate small and medium-sized pulmonary arteries, leading to dysregulation of angiogenesis. AECA: antiendothelial cell antibodies; RANTES: regulated upon activation, normal T cells expressed and secreted; CCL5: chemokine ligand 5; CX3CLL: chemokine ligand I; IL-1: interleukin-1; IL-6: interleukin-6; PDGF: platelet-derived growth factor. Reproduced from Dhala A. Pulmonary arterial hypertension in systemic lupus erythematosus: current status and future direction. Clinical and Developmental Immunology, 2012, Article ID 854941. doi: 10.1155/2012/854941. This article is licensed under a Creative Commons Attribution License, which permits unrestricted use, distribution, and reproduction, provided that the original work is properly cited (151).

Histopathological changes in patients with CTD-PAH vary depending on the underlying disease. The histopathology of SLE-PAH is highly specific and characterized by immune complex-mediated vasculitis and the deposition of immunoglobulins, complement components, and autoantibodies in the intima and media of pulmonary arterioles, accompanied by fibrinoid necrosis and lymphocytic infiltration (23, 33). Concurrent thrombotic arteriopathy presents as eccentric intimal fibrosis, intraluminal thrombosis with recanalization, and the absence of concentric laminar intimal fibrosis typical of SSc-PAH. Vascular remodeling includes endothelial and smooth muscle cell proliferation, medial hypertrophy, and the occasional formation of plexiform lesions. Pathologically, SLE-PAH can be classified into vasculitic, vasculopathic, and mixed subtypes, with the vasculitic subtype closely associated with SLE disease activity (33, 34) (Figure 3). In patients with SSc-PAH, hypoxemia and ischemia–reperfusion injury caused by inflammation and endothelial injury may promote cytokine release and aggravate pulmonary vascular remodeling, fibrosis, and intraluminal microthrombosis, ultimately leading to progressive increases in pulmonary vascular resistance, pulmonary artery pressure, and right ventricular pressure (35). Furthermore, positivity for lupus anticoagulants (LACs) is a major risk factor for venous thromboembolism and PAH in patients with SLE (36). PAH is relatively rare in patients with pSS. Existing studies suggest that the occurrence of pSS-related PAH may be associated with positive anti-SSA antibodies, anti-ribonucleoprotein (RNP) antibodies, and hypergammaglobulinemia (28). When RA co-occurs with pulmonary interstitial fibrosis, pulmonary vasculitis, infection, or bronchospasm, intimal thickening of pulmonary vessels may occur, thereby increasing the risk of elevated pulmonary artery pressure; patients with pulmonary parenchymal involvement are more likely to have pulmonary vasculitis, and their prognosis is often poor (37). A recent study revealed that 30 to 47% of patients with PM/DM may develop ILD, and patients with PM/DM often have concurrent pulmonary diseases such as aspiration pneumonia or acute or chronic respiratory failure. These pulmonary lesions may induce PAH (38).

## 4 Clinical characteristics of CTD-PAH

In the early stage of CTD-PAH, physical examination may not reveal any obvious abnormalities, and the slow progression of the disease is often overlooked by patients. When PAH patients seek medical attention, the most common symptom is shortness of breath after activity, which may be accompanied by chest tightness, chest pain, and coughing, indicating that PAH has progressed to the point of causing symptoms. When CTD-PAH patients engage in vigorous exercise, symptoms such as dizziness, presyncope, and syncope may occur, often suggesting that PAH is severe and has caused symptoms related to insufficient cardiac output (39). As pulmonary artery pressure increases to a level that cannot be compensated for by the right heart, patients may develop symptoms of right heart failure, such as edema in the lower extremities, abdominal distension, and loss of appetite. The most common clinical and physical symptoms of PAH are second heart sound accentuation caused by elevated pulmonary artery pressure and systolic murmur associated with tricuspid regurgitation (3). CTD patients who present with these symptoms during physical examination should be screened for PAH; however, not all CTD-PAH patients display these symptoms. Other signs often correspond to clinical symptoms. For example, if right heart failure occurs, jugular vein distension, hepatomegaly, edema in the lower extremities, and multiple serous cavity effusions may occur (1). Additionally, CTD patients may present with persistent hypoxia and peripheral cyanosis (37). Abnormal reticular capillary vascular lesions may appear in the small vessels of the skin, manifesting as capillary dilation or rupture, providing crucial clinical diagnostic evidence for CTD-PAH (11). Furthermore, medium-sized vessels may be affected, as reflected by the presence of Raynaud’s phenomenon, which is observed in 90% of SSc patients. However, Raynaud’s phenomenon is not a specific manifestation of CTD-PAH and occurs in both CTD-PAH patients and IPAH patients (40).

However, symptoms such as dyspnea and limited movement in CTD patients are not always caused by PAH (3). These symptoms must be strictly differentiated from symptoms indicative of other organ involvement commonly seen in CTD patients to avoid misdiagnosis and mistreatment. ILD is the most common pulmonary manifestation of CTD. Clinically, it is characterized mainly by progressive dyspnea and an irritating dry cough, with Velcro rales audible in the lungs. High-Resolution Computed Tomography (HRCT) shows interstitial infiltration and reticular or honeycomb changes, which are significantly different from symptoms caused by hemodynamic abnormalities in PAH patients (41, 42). Anemia is a common complication in CTD patients and is often caused by immune-mediated hematopoietic dysfunction or the impact of immunosuppressive therapy. Symptoms include not only dyspnea but also fatigue and pale complexion. Laboratory tests revealed decreased hemoglobin levels, which are not directly pathologically associated with PAH (43). Myopathy (such as muscle damage related to polymyositis/dermatomyositis) is characterized mainly by symmetrical proximal limb muscle weakness and myalgia. Limited movement results from muscle function impairment and is unrelated to pulmonary vascular and cardiopulmonary function abnormalities. Muscle enzyme spectrum tests can assist in differential diagnosis (44). Pericardial diseases (such as pericarditis and pericardial effusion related to systemic lupus erythematosus) may present with chest tightness and dyspnea and are often accompanied by precordial pain and pericardial friction rub. Echocardiography can reveal the location and degree of pericardial lesions, which are essentially different from the core feature of increased pulmonary vascular resistance in PAH patients (45). Cardiac involvement caused by CTD (such as myocarditis and valvular heart disease) often presents with dyspnea related to cardiac insufficiency, which can be clearly differentiated through electrocardiogram(ECG), echocardiography, and muscle enzyme spectrum tests (46, 47).

Therefore, for patients with CTD, clinicians should focus on identifying high-risk factors for PAH, strictly distinguishing them from other common causes, such as ILD, anemia, myopathy, pericardial disease, and cardiac involvement, and strengthening early screening for PAH to diagnose potential PAH in the subclinical stage before symptoms appear, thereby improving patient prognosis (48).

## 5 Screening for and evaluation of CTD-PAH

### 5.1 Early screening for CTD-PAH

To clarify the scope of different assessment strategies and avoid clinical confusion, A distinction should be drawn between diagnosis (symptoms, examinations, etc.) and screening, as well as “etiological screening.” Screening for CTD-PAH is applicable to CTD patients without a confirmed PAH diagnosis, while etiological screening is primarily used to identify potential CTD in newly diagnosed PAH patients. Diagnosis refers to confirming PAH through confirmatory examinations (e.g., RHC). Based on this classification, early CTD-PAH screening for patients with CTD but without a confirmed PAH diagnosis should include pulmonary function testing, ECG, and chest X-ray, and a composite screening model should be used to determine the probability of PAH. If the results strongly suggest PAH, RHC should be performed to obtain a definitive diagnosis (3).

#### 5.1.1 Etiological screening

Patients with PAH who are first seen in cardiology or respiratory departments must be screened for CTDs. Previous studies have indicated that CTD-PAH accounts for 25% of PAH cases (16, 35, 49). Therefore, all patients diagnosed with PAH should undergo routine screening for CTD through detailed medical history inquiries about symptoms such as joint swelling and pain, Raynaud’s phenomenon, purpuric rash, oral ulcers, alopecia, photosensitivity, dry mouth and eyes, parotid gland swelling, and tooth loss, as well as through comprehensive physical examinations to identify the presence of signs such as sausage-like fingers, digital ulcers, butterfly rash, rampant caries, and mirror tongue (2). Moreover, tests for antinuclear antibodies, anti-dsDNA antibodies, anti-ENA antibodies, and antiphospholipid antibodies should be performed (50). Different types of CTD-PAH have distinct clinical manifestations, treatment strategies, and prognoses. Rheumatologists and immunologists should be invited to assist in differential diagnosis to clarify the type of CTD and to assess the condition of the primary disease, thereby enabling the development of more accurate treatment plans (16).

#### 5.1.2 Pulmonary function

Pulmonary function parameters include forced vital capacity (FVC), total lung capacity (TLC), forced expiratory volume in one second (FEV1), forced expiratory volume in one second to forced vital capacity (FEV1/FVC) ratio, diffusion capacity for carbon monoxide (DLCO), and maximum expiratory flow at 75% forced expiratory volume (MEF75) (51). A sensitivity of 64–100% and a specificity of 32–96% were reported for the FVC/DLCO ratio in detecting PH or PAH across the reviewed studies (52, 53). Aithala et al. demonstrated that pulmonary function tests can be used to screen for CTD-PAH (40). Additionally, in 2009, Jing et al. reported that PAH is associated with peripheral airway obstruction and reduced diastolic pressure (54). Furthermore, systemic inflammation plays a significant role in the pathogenesis of PAH (55). Proinflammatory mediators may damage pulmonary arteries and airways simultaneously (56). Moreover, PAH is characterized by progressive pulmonary vascular remodeling and vasoconstriction, increased alveolar–capillary membrane thickness, and abnormal endothelial cell proliferation, which may impair airway function and consequently reduce the DLCO (54, 57, 58). Günther et al. suggested that a disproportionately low DLCO in SSc-PAH patients may be associated with pulmonary venous occlusive disease (59). However, it should be noted that a disproportionately low DLCO is a common manifestation in most SSc-PAH patients, which is mainly related to pulmonary vascular remodeling and alveolar-capillary membrane damage, and is not specifically indicative of PVOD.

#### 5.1.3 Chest X-ray/electrocardiogram

Chest X-ray and ECG are the simplest and most accessible examination types for patients with PAH. Compared with ultrasound, which is a subjective methodology, chest X-ray and ECG allow the presence of PAH to be assessed more objectively. The typical signs of PAH on chest X-ray include protrusion of the pulmonary artery segment and central pulmonary artery dilation (60). These manifestations are often not present in early-stage PAH patients (chest X-ray may be normal); thus, missed diagnoses are possible (61). The typical signs of PAH on ECG include pulmonary P waves, rightward deviation of the QRS electrical axis, right ventricular hypertrophy, right bundle branch block, and a prolonged QTc interval (62, 63).

#### 5.1.4 Composite screening algorithms

Given the limitations associated with single diagnostic modalities alone, approaches incorporating multiple assessments—including ECG, echocardiography, computed tomography, and pulmonary function testing—have been employed to increase the accuracy of PH detection. In the identification of PH or PAH in patients with SSc, such combined strategies have yielded sensitivities ranging from 87 to 100% and specificities between 48 and 92% (62, 64–66), as documented in previous research. Gladue and colleagues (65) revealed that compared with the use of any single test alone, the integrated use of echocardiography-derived right ventricular systolic pressure (RVSP) and key pulmonary function parameters (namely, the DLCO and the FVC/DLCO ratio) yielded superior sensitivity and negative predictive values (NPVs) for the diagnosis of PAH.

The DETECT algorithm represents a core, stepwise screening algorithm designed to identify SSc patients at high risk of PAH who warrant confirmatory RHC (3). In a prospective, multicenter DETECT investigation, a cohort of systemic sclerosis patients at high risk for PAH, defined by a DLCO < 60% and a disease duration longer than 3 years, were recruited, and all participants underwent standardized RHC (52). Among the included patients, 64% were categorized as World Health Organization functional class (WHO-FC) I or II. Additionally, a two-step algorithm for selecting candidates who require diagnostic RHC was developed. Step 1: The following variables were incorporated: the FVC/DLCO ratio, telangiectasia, anti-centromere antibody positivity, serum uric acid and NT-proBNP levels, and right axis deviation on ECG. Step 2: Two echocardiographic indicators were included: the right atrial area and the tricuspid regurgitation velocity (TRV). For the detection of PAH, this algorithm had a sensitivity of 96%, a specificity of 48%, a positive predictive value of 35%, and a negative predictive value of 98%. Later, single-center studies verified that the DETECT algorithm provides superior sensitivity and NPVs compared with echocardiography alone, even among patients with a DLCO ≥ 60% (67, 68). However, a recent *post hoc* analysis of the original DETECT population demonstrated reduced sensitivity when contemporary hemodynamic criteria for PAH were applied (69). Given its high referral rate (62%) and false-positive rate, further studies have been conducted to optimize the specificity and PPV of the DETECT algorithm. For example, Santaniello et al. (70) reported that integrating cardiopulmonary exercise testing (CPET) into the algorithm increased the specificity to 77.8% and the PPV to 63% while maintaining sensitivity. Similarly, Colalillo et al. (71) demonstrated that combining the TAPSE/sPAP ratio with the DETECT framework increased the PPV from 31 to 62%.

In contrast, the ASIG algorithm functions as a simplified risk stratification and first-tier assessment tool rather than a definitive screening pathway and is mainly used to triage patients for further testing (3). On the basis of a cohort of 49 SSc patients who underwent RHC, Thakkar and colleagues (70) developed the ASIG screening tool, which combines NT-proBNP and pulmonary function test results. The algorithm demonstrated a sensitivity of 94.1%, a specificity of 54.5%, a PPV of 61.5%, and an NPV of 92.3%. The results of this “first-tier” algorithm suggest that if either of 2 components is present (a DLCO <70% predicted with a FVC/DLCO ratio ≥ 1.8 AND/OR a NT-proBNP level > 210 pg./mL), the patient should undergo further evaluation with Transthoracic echocardiography (TTE), HRCT, ventilation-perfusion (V/Q) scanning, and the 6-min walk test (6MWT), and RHC should be performed in patients suspected of having PAH. Further tests for PAH are unnecessary in patients in whom both components are absent.

**Figure 4:**
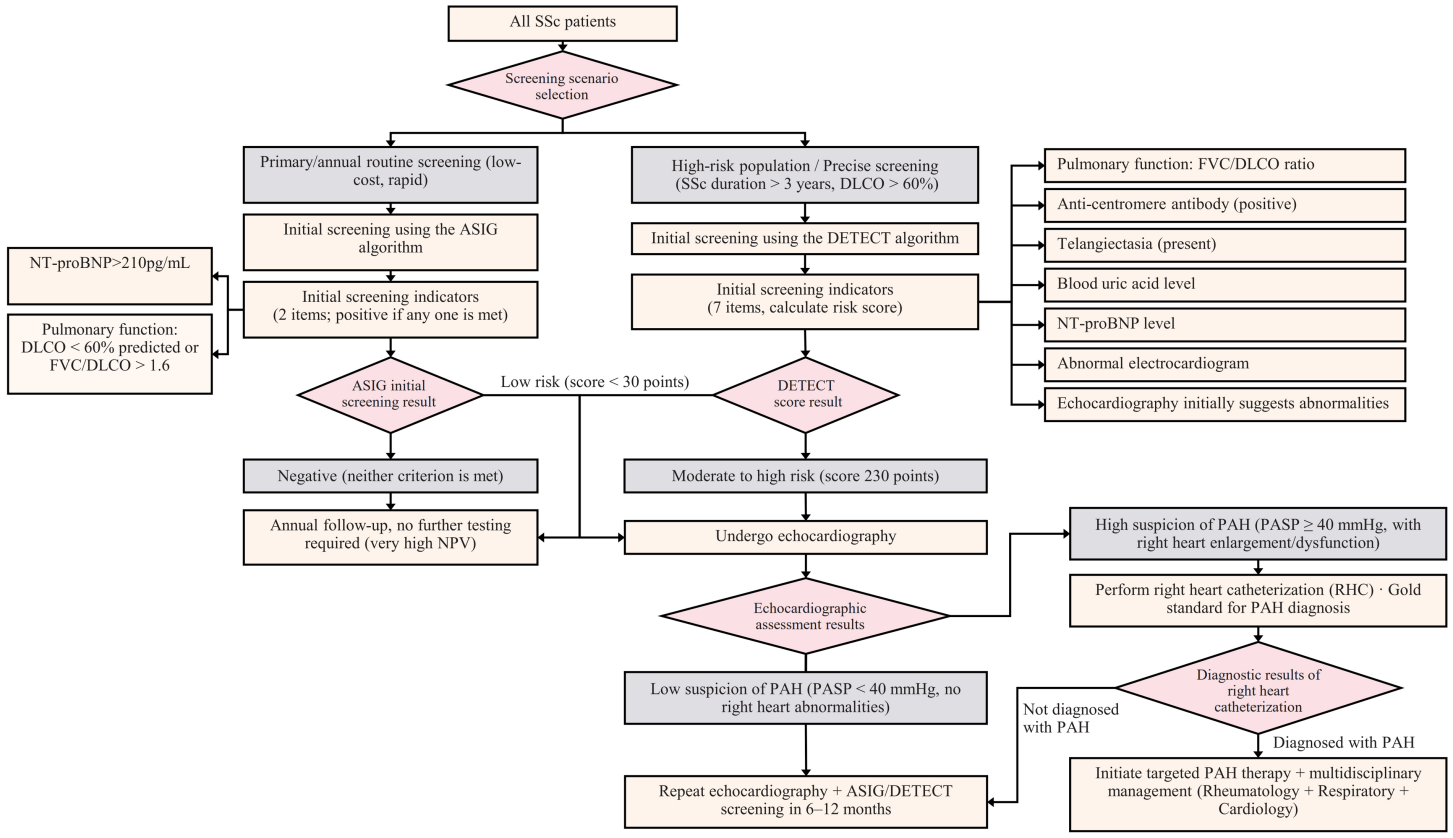
ASIG and DETECT algorithm-based screening pathways for PAH in patients with SSc. This figure is the original work of the authors and has not been published previously or submitted elsewhere.

In a comparative analysis of the DETECT, ASIG, and 2009 ESC/ERS algorithms for PAH screening in individuals with systemic sclerosis, Hao et al. (72) reported that both the DETECT and ASIG algorithms exhibited greater sensitivity (both 100%) than the ESC/ERS algorithm did (96.3%). In addition, compared with the DETECT algorithm, the ASIG algorithm showed superior specificity (54.5% vs. 35.3%) and positive predictive value (60% vs. 55.1%), as well as a lower referral rate. In a separate investigation, Vandecasteele and colleagues (73) compared the DETECT algorithm with the 2009 and 2015 ESC/ERS echocardiography-based screening criteria. All three approaches successfully identified all patients with PAH; however, the DETECT algorithm had the highest referral rate (30%) and the lowest PPV (6%). Notably, the DETECT algorithm was more likely to suggest right heart catheterization (93%) than the 2009 and 2015 ESC/ERS guidelines were (29 and 71%, respectively) among patients whose mean pulmonary arterial pressure ranged from 21 to 24 mmHg (Figure 4).

### 5.2 Assessment of CTD-PAH

Patients diagnosed with CTD-PAH should undergo comprehensive assessments to determine the severity and reversibility of the disease, which can guide the selection of appropriate treatments. For patients with CTDs, the activity and degree of disease-related damage should be evaluated on the basis of the disease type. For patients with PAH, assessments such as transthoracic echocardiography (74, 75), hemodynamic evaluations (76), anti-U1 ribonucleoprotein (U1-RNP) antibody level measurements (77), exercise tolerance assessments (40), and hemodynamic indicator measurements (78) should be conducted. Risk stratification should also be performed to adjust the treatment plan as needed during follow-up.

#### 5.2.1 Exercise tolerance assessment

All PAH patients should complete the WHO-FC and the 6-min walk test (2, 79). These are important indicators for assessing the severity of the disease and predicting survival in PAH patients. Changes in functional classification before and after treatment are also key indicators for evaluating treatment efficacy (50). The specific WHO-FC is similar to the NYHA cardiac function classification (Table 4); however, symptoms such as syncope associated with right heart failure are also included (3). The 6MWT is a valuable tool for functional evaluation, follow-up monitoring, and prognostic assessment in patients with CTD-PAH (40). In this test, patients walk as far as possible along a 30-meter straight corridor within 6 min, and the distance covered (6MWD) is recorded. Vital signs are closely monitored before and after exercise (80, 81). A shorter distance in the 6-min walk test is clinically significant for guiding treatment decisions. The 6MWD is significantly correlated with the prognosis of PAH patients (80, 81). The 6MWT is both reproducible and standardized and thus serves as an inexpensive and reusable method for assessing the physical capacity of patients. The time at which dyspnea or a decrease in oxygen saturation occurs can be determined in addition to the achievable distance^.^ (79). In numerous randomized controlled trials on PAH, the 6MWT has provided crucial reference indicators(95). While the 6MWT has demonstrated reliability in the assessment of IPAH, its validity in scleroderma assessment remains unclear, as many SSc patients experience musculoskeletal pain that prevents them from reaching dyspnea-limited distances (82).

**Table 4:** World Health Organization functional classification.

Classification	Standard
Grade I	The patient’s physical activity is not limited. Ordinary physical activity does not cause dyspnea, fatigue, chest pain, or near syncope.
Grade II	The patient has mild limitations of physical activity. No symptoms at rest, but ordinary physical activity results in dyspnea, fatigue, chest pain, or near syncope.
Grade III	The patient has marked limitations of physical activity. No symptoms at rest, but less than ordinary physical activity causes dyspnea, fatigue, chest pain, or near syncope.
Grade IV	The patient is unable to engage in any physical activity, with signs of right heart failure. Dyspnea and/or fatigue may occur at rest, and any physical activity will exacerbate the symptoms.

Reprinted from Vachiéry J. L., Simonneau G. Management of severe pulmonary arterial hypertension. Eur Respir Rev. (2010) 19:279–87. doi: 10.1183/09059180.00008010. This article is licensed under a Creative Commons Attribution License, which permits unrestricted use, distribution, and reproduction, provided that the original work is properly cited (157).

#### 5.2.2 Transthoracic echocardiography (TTE)

TTE is an essential tool for screening patients with suspected PAH and may also serve as an instrument for patient follow-up and monitoring treatment response (74, 75). Pulmonary artery systolic pressure and right ventricular size and function can be estimated by echocardiography. Echocardiography can also be used to assess valve function, identify valve stenosis or regurgitation, measure the size and function of cardiac chambers, and determine the presence and volume of pericardial effusion (83, 84). Tricuspid regurgitation is also important in the echocardiographic evaluation of PAH. Right ventricular dilatation, abnormal interventricular septal morphology and function, increased right ventricular wall thickness, and pulmonary artery trunk dilatation are also indicators of PAH (40). Compared with pulmonary function testing, TTE has greater sensitivity and specificity for diagnosing PAH (83). Notably, there is no definite correlation between pulmonary artery pressure estimated solely on the basis of the tricuspid regurgitation velocity and the severity of PAH. Clinicians must evaluate these indicators in combination with other symptoms and indicators. In addition, echocardiography can assist in excluding PH caused by congenital heart diseases (such as ventricular septal defects and patent ductus arteriosus) and left heart-related diseases (such as mitral stenosis and severe regurgitation) (85).

#### 5.2.3 Anti-U1-RNP antibodies

Anti-U1-RNP antibodies bind to the U1 small nuclear ribonucleoprotein (U1snRNP) autoantigen, forming a complex involved in the splicing of heterologous nuclear RNA into mRNA (86). PAH patients with higher anti-U1-RNP antibody levels are often younger at diagnosis, exhibit milder skin involvement, and more commonly present with swollen hands, Raynaud’s phenomenon, and joint pain (77). *In vitro* studies have demonstrated that anti-U1-RNP antibodies markedly increase the expression of endothelial leukocyte adhesion molecule-1 (ELAM-1), intercellular adhesion molecule-1 (ICAM-1), and the major histocompatibility complex (MHC) on human pulmonary endothelial cells. This leads to the formation of immune complexes, the proliferation of pulmonary vascular wall cells, and the occurrence of microvascular thrombosis. Ultimately, through immunological and inflammatory effects, pulmonary vascular stenosis and remodeling occur (87). Patients with immune vasculitis also exhibit susceptibility to vascular wall inflammation and vascular injury, which are correlated with upregulated ICAM-1 expression (88). Consequently, anti-U1-RNP antibodies may be involved in the pathogenesis of CTD-PAH.

Reports have indicated associations between the levels of anti-RNP antibodies and both SLE-PAH and pSS-PAH (89–91). Anti-U1-RNP antibody levels are higher in MCTD patients with PAH than in those without PAH; furthermore, anti-U1-RNP antibodies have been likely associated with the development of SLE-PAH and implicated in its pathogenesis. The vascular pathologies of SLE-PAH and MCTD-PAH are similar, with histological examination of the pulmonary arteries revealing intimal hyperplasia, medial thickening, reticular lesions, and focal microthrombi (8, 92). Thus, anti-U1-RNP antibodies may represent a marker for a distinct phenotype of CTD-PAH.

Studies indicate that CTD-PAH patients who are positive for anti-U1-RNP antibodies exhibit less severe cardiopulmonary impairment. For instance, a greater proportion of these patients are classified as WHO-FC I or II, whereas fewer are classified as class III or IV; furthermore, these patients walk greater distances in the 6MWT and have higher DLCO values. This may be attributable to the predominance of SLE-PAH and MCTD-PAH within the anti-U1-RNP antibody-positive population. Immunosuppressive therapy has been shown to alleviate symptoms and increase survival rates in patients with SLE-PAH or MCTD-PAH. Additionally, anti-U1-RNP antibody-positive patients tend to be younger (77). In summary, anti-U1-RNP antibody positivity may be correlated with increased survival rates in patients with CTD-PAH.

#### 5.2.4 Serological assessment

Serological assessments include assessments of BNP and NT-proBNP levels. Brain natriuretic peptide type B is reactively released by ventricular myocytes in response to increased wall tension and has been demonstrated to be an independent predictor of SSc-PAH (78). In a retrospective study of 138 outpatients with PAH, serum BNP levels were closely correlated with long-term patient prognosis. Furthermore, BNP levels are linearly related to hemodynamic parameters, right ventricular structural remodeling, exercise capacity, and the WHO-FC. Serum BNP can be used to assess patient prognosis and long-term right ventricular remodeling (76). In PAH patients, the level of N-terminal pro-brain natriuretic peptide (NT-proBNP) is correlated with pulmonary vascular disease severity and right ventricular dysfunction (93, 94). Compared with the level of BNP, the level of NT-proBNP is more significantly affected by patient age and renal function. Williams et al. demonstrated that the NT-proBNP threshold for predicting SSc-related PAH is 395 pg./mL (95). Furthermore, Mathai et al. reported that NT-proBNP levels were significantly higher in SSc-PAH patients than in IPAH patients (96). BNP and NT-proBNP levels reflect the severity of right ventricular dysfunction, and NT-proBNP levels are highly predictive of survival in patients with SSc-PAH (16). To assess disease status and guide treatment decisions in PAH patients, regular measurements of plasma BNP and NT-proBNP levels are needed at initial diagnosis and during follow-up (3).

#### 5.2.5 Hemodynamic evaluation

RHC is the most reliable method for assessing the severity of PAH. Elevated right atrial pressure (RAP), decreased cardiac output (CO), increased PVR, and reduced mixed venous oxygen saturation (SvO2) all indicate disease progression and poor prognosis in PAH patients. Owing to the invasive nature of RHC, regular RHC follow-ups are difficult to perform (97). RHC should be repeated in patients who exhibit poor responses to regular PAH treatment or before long-term reduction in the level of target drugs is implemented to effectively evaluate changes in PAH and assist in determining whether the treatment goal has been achieved or if treatment should be adjusted (97).

#### 5.2.6 Cardiac magnetic resonance/cardiopulmonary exercise Test

**Figure 5:**
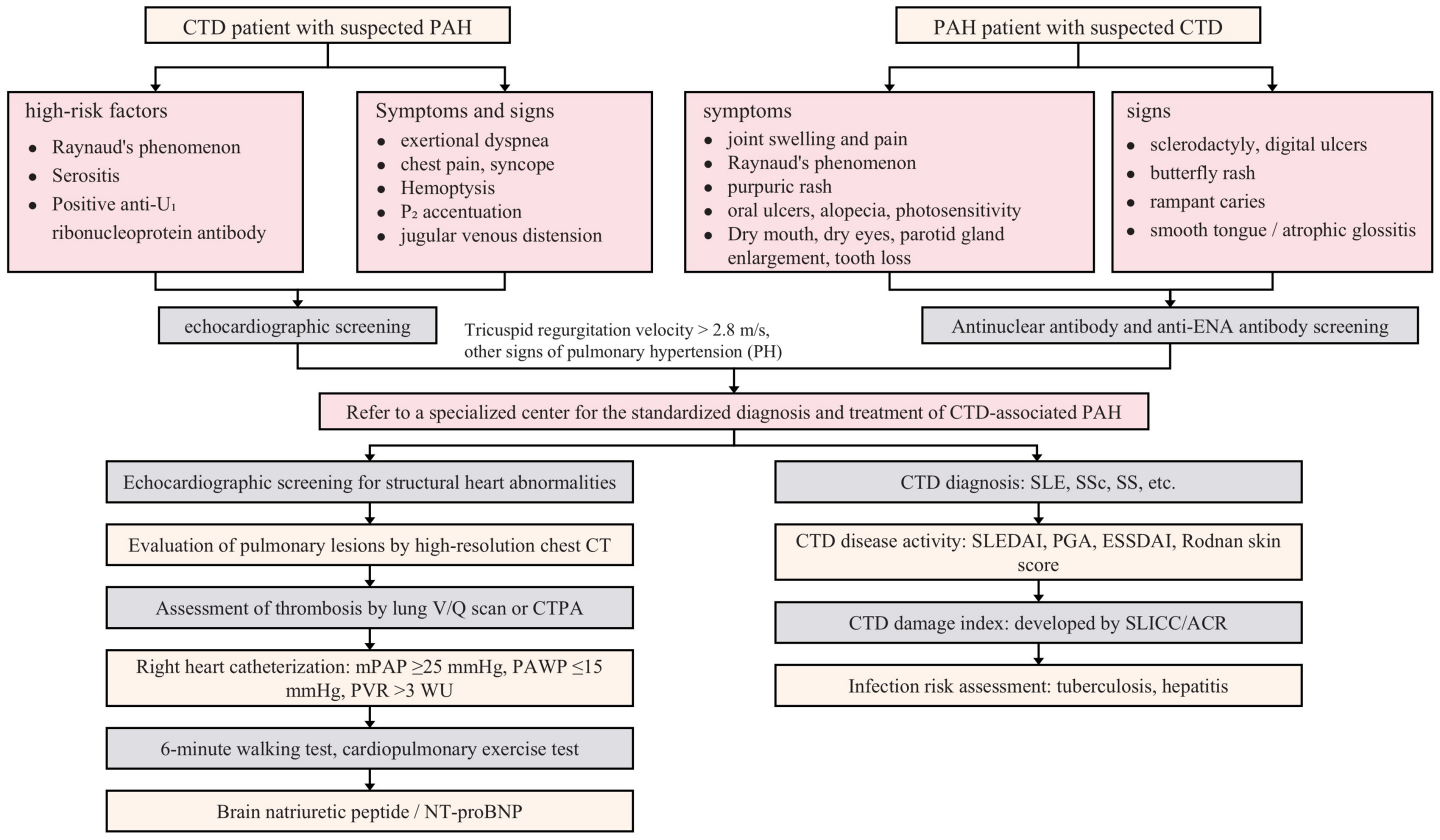
Screening and disease assessment for CTD-PAH. This figure is the original work of the authors and has not been published previously or submitted elsewhere.

Cardiac magnetic resonance (MR) imaging is currently the gold standard for evaluating right heart size, shape, and function, and this approach is highly repeatable (98, 99). The advantages of cardiac MRI include its noninvasive nature, lack of ionizing radiation, and high reproducibility. Its limitations include high cost, limited accessibility, and long examination time (97). The pulmonary artery-to-aorta diameter ratio, ventricular mass index (VMI), pulmonary artery velocity, and pulmonary artery distensibility are some cardiac MRI parameters used for PAH detection (99). In a study including SSc patients, a VMI > 0.56 had a sensitivity of 100% and a specificity of 70% for detecting PH (100). In the diagnosis of PAH in patients with CTDs, Rajaram et al. (70) reported a sensitivity of 85% and a specificity of 82% for a VMI > 0.45 and a sensitivity of 80% and a specificity of 78% for a pulmonary artery distensibility <15%. In another study, Hsu et al. (101) reported moderate sensitivity (68 and 57%) and specificity (57% for both) for the pulmonary artery diameter and maximum pulmonary artery velocity in detecting PH in SSc patients. A case–control study revealed that both the right ventricular free wall GLS (RVFW GLS) and the right ventricular ejection fraction (RVEF) measured on cardiac MR images have high sensitivity (84 and 95%, respectively) and specificity (77 and 84%, respectively) for detecting SSc-PAH (102). Exercise testing is an important objective and quantitative examination for evaluating cardiopulmonary reserve function and can be used to assess the treatment effect and prognosis of PAH patients. At diagnostic and treatment centers that meet the necessary requirements, assessments can be conducted on the basis of the actual characteristics of the patients (103) (Figure 5).

#### 5.2.7 Stratification of risk levels

Although risk stratification is currently the preferred assessment method for PAH, clinicians should be aware that any risk stratification method has limitations (2). Owing to the lack of systematic data (such as echocardiography and cardiac magnetic resonance images) in most cohort studies, relevant indicators for prognosis assessment are lacking; however, this does not indicate that the indicators are not related to prognosis (3). In addition, hemoptysis, pulmonary artery aneurysmal dilation, and arrhythmia, which are closely related to the severity of the disease, have not been included in previous studies, but clinicians should be aware of the risks associated with these complications (3, 104, 105) (Table 5). A recent study on Chinese CTD-PAH patients showed that the risk stratification model based on NT-proBNP, 6MWD and WHO-FC can effectively predict the prognosis of patients, with a 1-year survival rate of 92% in low-risk patients and 65% in high-risk patients (106). In Chinese patients, SLE-PAH patients often have a better prognosis than SSc-PAH patients in the same risk stratum, which may be related to the better response of SLE-PAH to immunosuppressive therapy (15).

**Table 5:** Simplified risk stratification of PAH.

Prognostic factors		Low risk	Medium risk	High risk
A	WHO functional classification	Grade I, Grade II	Grade III	Grade IV
B	6-min walking distance	> 440 m	165 ~ 440 m	< 165 m
C	Plasma BNP/NT-proBNP levels or RAP	BNP < 50 ng/L, NTproBNP < 300 ng/L or RAP < 8 mmHg	BNP50 ~ 300 ng/L, NTproBNP 300 ~ 1,400 ng/L or RAP8 ~ 14 mmHg	BNP > 300 ng/L, NT_proBNP > 1,400 ng/L or RAP > 14 mmHg
D	Hemodynamic indicators	CI ≥ 2.5 L ·min^−1^ ·m^−2^, SO_2_ > 65%	CI 2.0 ~ 2.4 L ·min^−1^ ·m^−2^ SO_2_ 60% ~ 65%	CI < 2.0 L ·min^−1^ ·m^−2^ SO_2_ < 60%

Reproduced from Raina A., Humbert M. Risk assessment in pulmonary arterial hypertension. Eur Respir Rev. 2016; 25 (127):390–398. doi: 10.1183/16000617.0077-2016. This article is licensed under a Creative Commons Attribution License, which permits unrestricted use, distribution, and reproduction, provided that the original work is properly cited (102).

## 6 Treatment of CTD-PAH

### 6.1 General treatment

In accordance with recent joint ESC/ERS clinical guidelines, the treatment protocol for CTD-PAH follows the same stepwise intensified pharmacological regimen as that used for IPAH (3). Referral to a specialized PH center is recommended when possible, with general supportive care including oxygen supplementation, mild exercise training, and individualized diuretic therapy (102).

A meta-analysis assessing anticoagulation requirements in CTD-PAH patients revealed no reduction in mortality among those who received oral anticoagulants (107). In a retrospective study involving 663 non-idiopathic PAH patients, including 168 CTD-PAH patients, the response of the patients to acute vasodilator testing and long-term calcium channel blocker (CCB) therapy was evaluated. The findings revealed that 10.1% (*n* = 17) of the CTD-PAH patients exhibited positive acute vasodilator responses, whereas only one patient (0.6%) exhibited a sustained long-term response to CCB treatment. After one year of therapy, this patient displayed improved hemodynamic parameters and clinical symptoms, with a stable New York Heart Association (NYHA) functional class I–II status (79).

### 6.2 Immunotherapy for CTD-PAH

**Figure 6:**
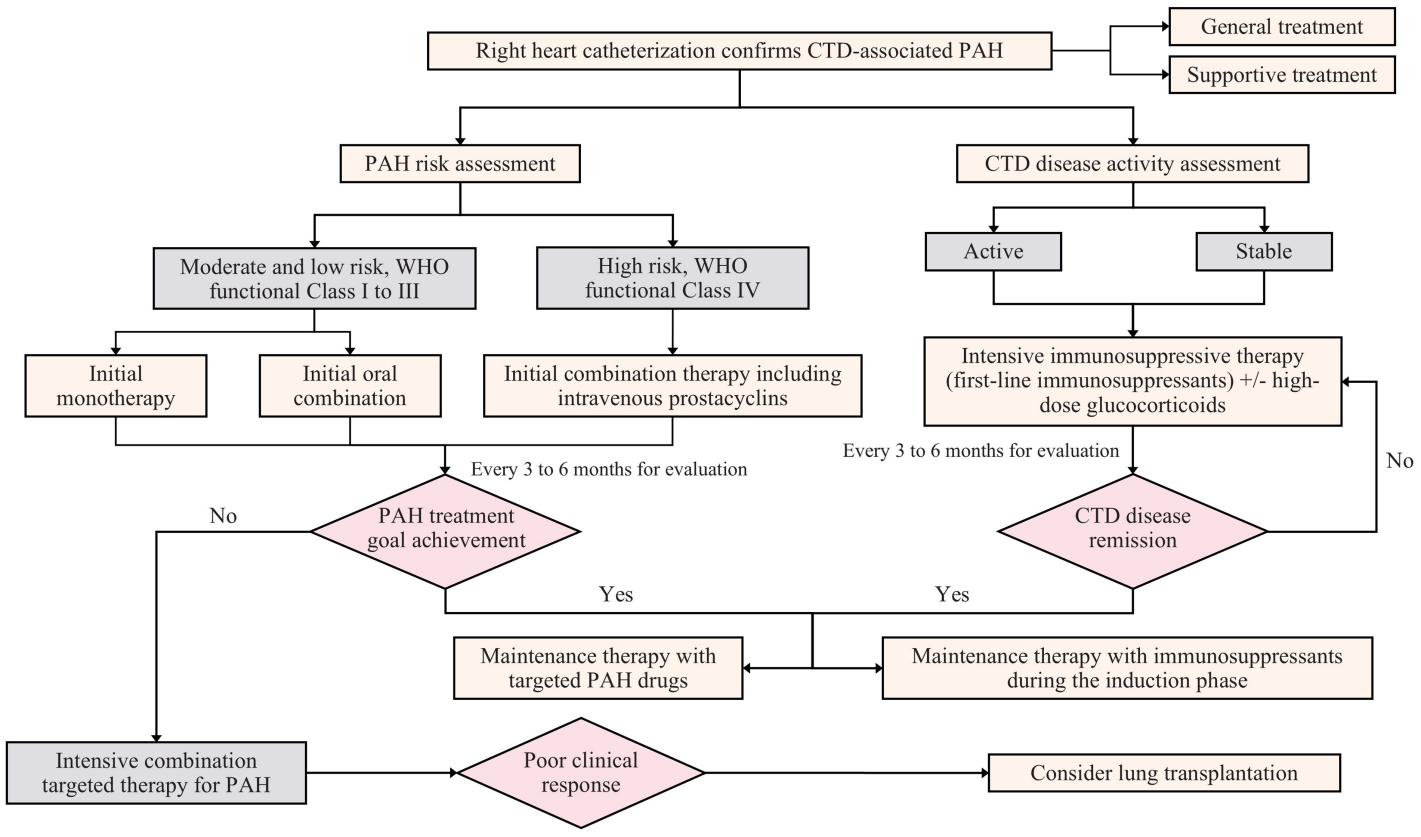
Diagnosis of and treatment strategies for CTD-PAH. This figure is the original work of the authors and has not been published previously or submitted elsewhere.

Given the established role of inflammatory and immune mechanisms in the pathogenesis of CTD-PAH, immunosuppressive therapies are employed across various CTD-PAH subtypes. Prior studies have suggested that immunosuppressive therapy may improve survival in patients with SLE-PAH or MCTD-PAH (108, 109). Jais and colleagues reported that among patients with SLE-PAH or MCTD-PAH who received combination therapy, including cyclophosphamide and glucocorticoids, nearly half exhibited clinical improvement in terms of cardiopulmonary function and hemodynamic parameters (109). Subsequent studies have corroborated these findings, demonstrating improvements in cardiopulmonary function, hemodynamics, and survival with immunosuppressive therapy in CTD-PAH patients (108, 110, 111). A prospective study also revealed that 46% of CTD-PAH patients treated with cyclophosphamide combined with pulmonary vasodilators experienced improvements in mean pulmonary artery pressure, NYHA functional class, and 6MWD (40). Furthermore, Sanchez et al. reported that compared with nonresponders, patients who responded to immunosuppressive therapy had a lower baseline NYHA functional class and less severe pulmonary hemodynamics (108, 111) (Figure 6). In contrast, in patients with SSc-PAH, glucocorticoids and immunosuppressants do not consistently improve symptoms, hemodynamics, or prognosis. Therefore, the decision to use glucocorticoids or immunosuppressants in patients with SSc-PAH should be individualized on the basis of the stage of SSc and the presence of other organ involvement (112, 113).

### 6.3 Targeted therapy for CTD-PAH

The management of patients with CTD-PAH should be guided by risk stratification, which dictates the initial choice of targeted therapy, either as monotherapy or combination therapy (114). Furthermore, treatment plans should be reassessed and adjusted during regular follow-up on the basis of the patient’s evolving risk status, with the ultimate goal of achieving and maintaining a low-risk profile (115). Targeted therapies represent major advances in PAH treatment and have significantly improved patient prognosis. Numerous targeted therapies have demonstrated efficacy in CTD-PAH patients, both as initial monotherapies and as part of combination regimens for those who fail to reach treatment goals (116). In Western countries, phosphodiesterase-5 inhibitors (PDE5Is), such as sildenafil, tadalafil, and vardenafil, are approved for PAH treatment (117). Although PDE5Is are not yet officially approved for PAH treatment in China, they are widely used in clinical practice because of their proven efficacy and favorable cost-effectiveness profile, effectively serving as a first-line treatment option in this region (118) (Table 6).

**Table 6:** Types, recommended usage, and adverse reactions of targeted therapies for PAH.

Medicine	Indication	Recommended usage (For adults)	Adverse reactions
Endothelin receptor antagonists			
Bosentan	PAH	Oral administration: 62.5–125 mg twice daily.	Elevated transaminase, peripheral edema, anemia
Ambrisentan	PAH	Oral administration: 5–10 mg once daily.	Headache, peripheral edema, anemia
Macitentan	PAH	Oral administration: 10 mg once daily.	Anemia
Prostacyclin analogs			
lloprost	PAH	Nebulization: 10–20 μg per time, 6–9 times daily. Requires a special nebulizer.	Facial flushing, hypotension, cough, headache
Treprostinil	PAH	Titration required: subcutaneous injection or continuous intravenous infusion. Starting dose: 1.25 ng·kg^−1^·min^−1^, which can be gradually increased to 20–40 ng·kg^−1^·min^−1^.	Injection site pain, headache, gastrointestinal symptoms
Beraprost	None	Oral administration: 40–120 μg four times daily.	Headache, flushing
Prostacyclin receptor agonists			
Selexipag	PAH	Titration required: oral administration, starting at 200 μg twice daily. Increase by 200 μg weekly until the tolerated dose is reached. Maximum dose: 1600 μg twice daily.	Headache, gastrointestinal symptoms, jaw pain
5-Phosphodiesterase inhibitors			
Sildenafil	None	Oral administration: 20–80 mg three times daily.	Facial flushing, visual disturbances
Tadalafil	None	Oral administration: 10–40 mg once daily.	Hot flashes, myalgia
Vardenafil	None	Oral administration: 5–10 mg twice daily.	Hot flashes, myalgia
Guanylate cyclase agonists			
Riociguat	PAH and CTEPH	Titration required: oral administration, starting at 1 mg three times daily Increase the dosage every 2 weeks by 0.5 mg each time, gradually up to the maximum tolerated dose. The maximum dose is 2.5 mg three times daily.	Hypotension, gastrointestinal symptoms, headache
Activin receptor type IIA-Fc (ActRIIA-Fc) fusion protein			
Sotatercept	PAH	Recommended starting dose: 0.3 mg/kg, subcutaneously every 3 weeks; Recommended target dose: 0.7 mg/kg, subcutaneously every 3 weeks.	Headache, nosebleed, rash, telangiectasia, diarrhea, dizziness, and erythema

This table is the original work of the authors and has not been published previously or submitted elsewhere.

#### 6.3.1 Prostacyclin (PGI₂) Analog and prostacyclin receptor agonists

PAH patients exhibit deficiencies in prostacyclin synthase, resulting in suppressed prostacyclin production. This deficiency predisposes patients to thrombosis, vasoconstriction, and vascular smooth muscle proliferation (39). Treatment with synthetic prostacyclins and their analogs can reverse these changes, as these agents have potent vasodilatory and antiproliferative effects (39). However, prostaglandin derivatives may induce side effects such as headaches, facial flushing, jaw pain, and diarrhea (119).

#### 6.3.2 Endothelin receptor antagonists (ERAs)

Endothelin-1 (ET-1) is a peptide whose secretion is increased in PAH patients. Two endothelin receptors, endothelin receptor A (ETA) and endothelin receptor B (ETB), play crucial roles in PAH. ET-1 acts on the vascular endothelium and vascular smooth muscle, resulting in vasoconstriction and vascular endothelial hyperplasia, respectively (39). Bosentan is an endothelin receptor antagonist. However, approximately 10% of patients exhibit elevated liver transaminase levels following its use. This effect is reversible; hence, it is recommended that patients receiving bosentan undergo monthly liver function tests^.^ (27). Additionally, ambrisentan causes elevated liver transaminase levels in only 0.8–3% of patients, making it suitable for those who are unable to take bosentan because of the resulting abnormal liver function (120).

#### 6.3.3 Phosphodiesterase-5 inhibitors (PDE-5Is)

Nitric oxide (NO) is a critical vasodilator. It exerts its vasodilatory effects by maintaining intracellular cyclic guanosine monophosphate (cGMP) levels in vascular smooth muscle cells (51). In patients with PAH, reduced NO bioavailability contributes to abnormal vascular remodeling and cellular proliferation within the pulmonary vasculature. Phosphodiesterase-5 (PDE-5) inhibitors increase cGMP levels by inhibiting its degradation by PDE-5 enzymes, thereby promoting pulmonary vasodilation (121, 122). Sildenafil, the most extensively studied agent in this class, improves hemodynamics and clinical symptoms when administered orally, with trials demonstrating favorable outcomes and enhanced functional status (39, 40). Additionally, tadalafil, another PDE-5 inhibitor, has been shown to improve patients’ exercise capacity and hemodynamics (123). Compared with monotherapy, initial combination therapy with ambrisentan and tadalafil significantly reduces the incidence of adverse outcomes in patients (123, 124). Current guidelines recommend combination therapy for patients who fail to respond adequately or who experience disease progression while on monotherapy (125), as these drugs may exert synergistic effects through multiple targeted pathways, supporting the rationale for combination therapy.

#### 6.3.4 Guanylate cyclase agonists

Soluble guanylate cyclase (sGC) is also an important vasodilator (126). The mechanism of action of guanylate cyclase agonists in vasodilation involves the direct activation of sGC in pulmonary vascular smooth muscle cells without relying on NO. This promotes the conversion of guanosine triphosphate (GTP) to cGMP and increases intracellular cGMP concentration (127). Elevated cGMP levels inhibit pulmonary vascular smooth muscle cell contraction, suppress vascular remodeling and excessive cell proliferation, reduce pulmonary vascular resistance, and improve right heart function (127–129).

The efficacy of riociguat, both as a monotherapy and in combination with endothelin receptor antagonists (ERAs), has been investigated in patients with PAH. The RESPITE study demonstrated that 61 patients with an insufficient response to phosphodiesterase type 5 inhibitor (PDE5i) therapy showed improvements in exercise capacity and hemodynamics following riociguat treatment (130). Furthermore, the REPLACE study revealed that compared with patients who continued PDE5i therapy, patients who switched from PDE5i therapy to riociguat demonstrated significant improvements in their 6-min walk distance (6MWD), WHO-FC, and N-terminal pro-B-type natriuretic peptide (NT-proBNP) levels and a reduced incidence of clinical worsening events (131). The latest European Society of Cardiology (ESC)/European Respiratory Society (ERS) guidelines support the potential therapeutic benefits of this conversion treatment in the management of PAH (3).

#### 6.3.5 Prostacyclin IP receptor agonists

Prostacyclin IP receptor agonists represent another important class of targeted therapies for CTD-PAH. By selectively binding to prostacyclin (IP) receptors on pulmonary vascular smooth muscle cells and endothelial cells, these agents activate the Gs-AC-cAMP-PKA signaling pathway, leading to an increase in intracellular cyclic adenosine monophosphate (cAMP) levels. This cascade of events results in multiple beneficial effects, including the inhibition of pulmonary vascular smooth muscle cell contraction and abnormal proliferation, the suppression of vascular wall fibrosis and vascular remodeling, and the exertion of anti-inflammatory and anti-platelet aggregation properties. Collectively, these actions reduce the risk of *in situ* thrombosis within the pulmonary vasculature and, most importantly, lead to a decrease in pulmonary vascular resistance and a reduction in right heart afterload (132, 133).

The phase 3 GRIPHON trial investigated selexipag at its maximum tolerated dose in patients with PAH. This study included 334 patients with CTD-PAH, of whom 170 had SSc-PAH (77 in the treatment group and 93 in the placebo group). Enrolled patients were either treatment-naïve or were receiving a stable dose of PDE5 inhibitors, ERAs, or a combination of both. The results confirmed that selexipag at the maximum tolerated dose was well tolerated and led to a 44% reduction in the composite endpoint of morbidity and mortality in the treatment group (134).

#### 6.3.6 Activin receptor type IIA-fc (ActRIIA-fc) fusion protein

Bone morphogenetic protein receptor type II (BMPR-II) is a member of the transforming growth factor-beta (TGF-β) superfamily; together with activin receptor type IIA (ActR-IIA), it plays crucial roles—both anti-proliferative and pro-proliferative—in the normal growth of pulmonary arteries (135). An imbalance between the intracellular SMAD2/3 and SMAD1/5/8 signaling pathways is widely considered to be a significant contributing factor to the pathogenesis of PAH (136). Sotatercept is an ActR-IIA fusion protein, constructed by fusing the Fc domain of human immunoglobulin G1 (IgG1) with the extracellular domain of ActR-IIA; acting as an activin signaling inhibitor, it binds to activin A and other ligands within the TGF-β superfamily (137). Sotatercept regulates the proliferation and inhibition of pulmonary vasculature by restoring the balance between pro-proliferative (ActR-IIA/SMAD2/3) and anti-proliferative (BMPR-II/SMAD1/5/8) signaling pathways.

The STELLAR study (138) was a global, multicenter, double-blind, randomized, placebo-controlled phase III clinical trial (NCT04576988) designed to confirm the safety and clinical efficacy of sotatercept in addition to stable background therapy (monotherapy, dual therapy, or triple therapy with PAH-targeting drugs). The trial enrolled 323 patients with PAH of WHO-FC grade II or III. Patients were randomly assigned 1:1 to the sotatercept group (163 patients) and the placebo group (160 patients). Sotatercept was started at a dose of 0.3 mg/kg and gradually increased to a target dose of 0.7 mg/kg for 24 weeks. The primary clinical endpoint of the trial was the change in 6MWD from baseline at week 24. Secondary clinical endpoints included PVR, changes in NT-proBNP levels, improvement in WHO-FC, time to death or clinical deterioration, and PAH-SYMPACT score. The results showed that, regarding the primary clinical endpoint, the median change in 6MWD from baseline at week 24 was 34.4 meters (95% CI: 33.0–35.5) in the sotatercept group and 1.0 meters (95% CI: −0.3–3.5) in the placebo group. Hodges-Lehmann estimation showed a difference of 40.8 meters (95% CI: 27.5–54.1, *p* < 0.001) in the sotatercept group. Regarding secondary clinical endpoints, patients showed significant improvements in PVR, NT-proBNP levels, and WHO-FC. Of particular note, sotatercept reduced the risk of death or clinical deterioration by 84%, with 42 out of 160 patients in the placebo group experiencing death or clinical deterioration, compared to only 9 out of 163 patients in the sotatercept group. Furthermore, sotatercept demonstrated a significant advantage in improving multiple key indicators associated with PAH patients (6MWD ≥ 30 m, NT-proBNP decrease ≥30% or <300 ng/L, WHO-FC improvement or maintenance of WHO-FC grade II), with approximately four times more patients achieving simultaneous improvement in multiple indicators at week 24 (38.9% vs. 10.1%) compared to the placebo group. The mortality rate in the sotatercept group (1.2%) was slightly lower than that in the placebo group (4.4%) (138). Thrombocytopenia, elevated hemoglobin levels, and bleeding were the most common safety issues in the sotatercept group, and most of these events could be controlled by taking appropriate measures.

Subsequently, McLaughlin et al. (139) conducted an in-depth analysis of the STELLAR study data using a Markov-type model to evaluate and project the long-term clinical impact of sotatercept. The results indicate that sotatercept can extend the life expectancy of patients with by approximately 12 years, while also significantly reducing the risks of hospitalization and heart/lung transplantation. Whether sotatercept can achieve the model-projected outcomes requires further validation through real-world studies.

## 7 Follow-up and risk assessment for CTD-PAH

Regular follow-up visits are beneficial for controlling the condition and improving the prognosis of patients with CTD-PAH (3). CTD-PAH patients should be referred to specialized centers for follow-up management (140). CTD-PAH patients should be encouraged to follow up regularly and take medications as prescribed to achieve dual targets attainment in the shortest possible time. The frequency of follow-up visits should be adjusted on the basis of the patient’s condition: When the activity of the CTD (initial onset or recurrence) or immunosuppressive treatment is not stable, follow-up visits should be conducted every 1 to 3 months. Once the CTD is in remission and PAH targets are achieved, the follow-up interval can be adjusted to every 3 to 6 months (112).

During follow-up visits, clinicians should still conduct comprehensive assessments of both CTD and PAH and optimize follow-up management by combining the DETECT and ASIG algorithms, which are commonly used tools for subsequent follow-up and risk assessment of CTD-PAH (3). In particular, the activity, organ-related damage, and complications of CTD should be evaluated. Treatment plans should be adjusted on the basis of individual patient characteristics and their overall condition at follow-up visits, with the goal of maintaining low or no disease activity and reducing the occurrence of complications (3, 51). Additionally, complete evaluations, such as the WHO-FC, 6-min walk test, BNP/NT-proBNP level measurements, and echocardiography, should be performed. In addition, the DETECT algorithm and ASIG algorithm can be used in combination to assist in assessment: During the follow-up process, the core parameters of the DETECT algorithm (such as BNP/NT-proBNP level measurements, the DLCO, and ultrasound indicators) can still be used as dynamic monitoring tools to help identify the risk of early deterioration. It is particularly suitable for follow-up screening of PAH in patients with high-risk CTD subtypes such as SSc (11); the ASIG algorithm is more convenient and practical and can assist in determining disease progression trends. Therefore, it is more operable in scenarios with limited resources or in routine follow-up (141). Both are superior to the simple ESC/ERS ultrasound screening strategy and can be used as supplementary tools in the long-term management of CTD-PAH, helping clinicians make more precise decisions on follow-up frequency and providing a reliable basis for adjusting treatment plans and repeating invasive tests (142). Clinicians should perform risk stratification for PAH. The goal should be to achieve and maintain a low-risk classification of PAH (143). If patients have not reached the target after initial treatment, intensified targeted drug therapy should be considered (2, 3). If patients have a low-risk classification, drug discontinuation should not be considered immediately. A low-risk state should be maintained for at least one year, and only when the overall condition is stable and improving should the gradual reduction or discontinuation of targeted drugs be considered (144). When the cause of disease exacerbation is unknown or if treatment targets have not yet been achieved, right heart catheterization may be considered (145). It is crucial to avoid relying solely on echocardiographic estimates of pulmonary artery pressure to assess PAH, and the importance of comprehensive PAH assessments should be strongly emphasized (3).

## 8 Prognosis of CTD-PAH

PAH contributes to a poor prognosis in CTD patients (106). The prognosis of CTD-PAH depends on two key aspects. (1) The severity of PAH: The risk stratification strategy, DETECT algorithm, and ASIG algorithm are good methods for predicting the prognosis of CTD-PAH. (2) The specific type of CTD: The prognosis varies among patients with different types of CTDs. Among them, the prognosis of SLE and pSS-PAH is the best (15). Current cohort studies suggest that the 5-year survival rates of patients with SLE and pSS-PAH are 72.9 and 79%, respectively (146). The prognosis of SSc-PAH is slightly worse, with a 5-year survival rate of approximately 63% (146). This may be related to differences in the pathogenesis of the underlying CTD (147). With the standardization of PAH diagnosis and treatment approaches and the introduction of more targeted drugs at lower prices, the long-term prognosis of CTD-PAH patients is expected to improve in the future.

## 9 Summary

Although some studies have investigated the clinical manifestations, laboratory parameters, and survival prognosis of patients with CTD-PAH, most investigations have been performed in Western countries. Currently, data pertaining to the Han Chinese population remain scarce. Therefore, further research is needed to validate the clinical characteristics and prognostic factors of CTD-PAH in the Han Chinese population, with the aim of providing a basis for improving the prognosis of patients with CTD-PAH.
